# The influence of digital platform on the implementation of corporate social responsibility: from the perspective of environmental science development to explore its potential role in public health

**DOI:** 10.3389/fpubh.2024.1343546

**Published:** 2024-04-22

**Authors:** Mansi Wang, Renmiao Yuan, Xin Guan, Zeyu Wang, Yanzhao Zeng, Tao Liu

**Affiliations:** ^1^School of Management, Guangzhou University, Guangzhou, China; ^2^Guangzhou Xinhua University, Dongguan, China; ^3^School of Public Administration, Guangzhou University, Guangzhou, China; ^4^School of Economics and Statistics, Guangzhou University, Guangzhou, China; ^5^School of Journalism and Communication, Guangzhou University, Guangzhou, China

**Keywords:** digital platform, corporate environmental protection behavior, corporate social responsibility, sustainable development, intermediary analysis

## Abstract

**Introduction:**

This paper aims to explore the intersection of corporate social responsibility (CSR) and public health within the context of digital platforms. Specifically, the paper explores the impact of digital platforms on the sustainable development practices of enterprises, seeking to comprehend how these platforms influence the implementation of environmental protection policies, resource management, and social responsibility initiatives.

**Methods:**

To assess the impact of digital platforms on corporate environmental behavior, we conducted a questionnaire survey targeting employees in private enterprises. This survey aimed to evaluate the relationship between the adoption of digital platforms and the implementation of environmental protection policies and practices.

**Results:**

Analysis of the survey responses revealed a significant positive correlation between the use of digital platforms and the environmental protection behavior of enterprises ( r=0.523;p<0.001 ), Moreover, the presence of innovative environmental protection technologies on these platforms was found to positively influence the enforcement of environmental policies, with a calculated impact ratio of ( a∗b/c=55.31% ). An intermediary analysis highlighted that environmental innovation technology plays a mediating role in this process. Additionally, adjustment analysis showed that enterprises of various sizes and industries respond differently to digital platforms, indicating the need for tailored environmental policies

**Discussion:**

These findings underscore the pivotal role of digital platforms in enhancing CSR efforts and public health by fostering improved environmental practices among corporations. The mediating effect of environmental innovation technologies suggests that digital platforms not only facilitate direct environmental actions but also enhance the efficiency and effectiveness of such initiatives through technological advances. The variability in response by different enterprises points to the importance of customizable strategies in policy formulation. By offering empirical evidence of digital platforms’ potential to advance CSR and public health through environmental initiatives, this paper contributes to the ongoing dialogue on sustainable development goals. It provides practical insights for enterprises and policy implications for governments striving to craft more effective environmental policies and strategies.

## 1 Introduction

Global environmental issues have gained prominence in today’s society, raising a great deal of concern. Environmental challenges such as climate change, resource depletion and ecosystem destruction threaten the sustainable development of the earth and the survival of mankind (1, 2). In this context, enterprises not only need to find a balance between economic interests and environmental protection, but also need to hypothesize social responsibilities and contribute to sustainable development (3). As a tool for information dissemination, cooperation and interaction, and resource integration, digital platform is regarded as an emerging force that may have a far-reaching impact on corporate environmental protection behavior and social responsibility (4). In the past decades, corporate social responsibility (CSR) has become an important part of business practice. Enterprises no longer only pay attention to economic performance, but link their economic activities with social and environmental issues to ensure sustainable development (5–7). Meanwhile, the rise of digital platform has changed the interaction between enterprises and their stakeholders, providing enterprises with more opportunities to disseminate environmental information, cooperate to solve environmental problems, and supervise their environmental protection behavior (8). However, despite these potential opportunities, there are still many unknown factors about the actual impact of digital platforms on corporate environmental behavior and social responsibility (9).

In recent years, with the rapid development of digital technology, digital platform has become an important force to promote social change. Especially in corporate social responsibility and public health, the role of digital platform has become increasingly prominent. Early studies such as Wang et al. (10) have pointed out that digital transformation can promote enterprises to implement environmental protection policies and social responsibility plans more efficiently. However, there is still a lack of existing literature on how the digital platform affects the sustainable practice of enterprises under the guidance of the development of environmental science, especially the contribution to public health. At present, digital platform plays a vital role in the practice of CSR. Through digital means, enterprises can manage resources more effectively, improve energy efficiency, reduce carbon emissions and other environmental protection behaviors. Taking an energy company as an example, the company uses digital platform to implement intelligent energy management system, monitor energy usage, and optimize energy distribution, thus reducing energy waste and improving energy utilization efficiency. Through digital monitoring and data analysis, enterprises can know the energy consumption in real time, adjust production plans in time to reduce carbon emissions, and realize green production. These measures not only help enterprises to comply with environmental laws and regulations and fulfill their social responsibilities, but also bring them economic benefits and brand reputation. Looking forward to the future, the potential of digital platform lies in promoting enterprises to achieve sustainable development goals and promoting environmental protection behavior and social responsibility practice to a higher level. The continuous innovation and application of digital technology will provide more environmental protection solutions and tools for enterprises and support the realization of environmentally friendly production. However, the digital platform also faces some challenges, such as data privacy protection and information security risks, which need to be effectively controlled. Meanwhile, in the process of digital transformation, enterprises may face challenges in technology upgrading and talent training, and it is necessary to strengthen their understanding and application ability of digital technology. Considering the development perspective of environmental science, the relationship between digital platform and CSR is very important. Through the application of digital platform, enterprises can better practice environmental protection behavior, promote sustainable development, and integrate social responsibility into all aspects of business operations. The in-depth discussion of this relationship fills the gap in the existing research and provides new ideas and viewpoints for the related influence in the field of public health. By combining the concepts of digital platform, environmental science and CSR, future research will help to better explore the potential role of digital platform in CSR and public health, and promote the development of enterprises in a more sustainable and socially responsible direction. Therefore, this paper attempts to fill this knowledge gap and explore the subject through empirical research. Specifically, this paper uses the methods of descriptive statistical analysis, correlation analysis and hypothesis test analysis to evaluate the relationship between the use of digital platforms and corporate environmental behavior, investigates the impact of digital platforms on CSR policies and practices, explores the intermediary variables and moderating variables between digital platforms and corporate environmental behavior, and compares the differences in the impact of digital platforms on corporate environmental behavior and social responsibility between different industries and geographical regions. This paper deeply discusses the important role of digital platform in enterprise operation and the possible positive impact of corporate social responsibility on public health and environmental protection. With the acceleration of digital transformation, enterprises increasingly rely on digital platforms to optimize their operational efficiency and market competitiveness, which provides new opportunities and challenges for enterprises to fulfill their social and environmental responsibilities. By revealing how the digital platform can help enterprises to better implement CSR strategy, and then have a positive impact on environmental protection, this paper aims to provide policy makers and business managers with empirical insights and suggestions to promote the realization of sustainable development goals.

In order to achieve the above research objectives, this paper adopts various research methods, including quantitative questionnaire survey, to collect relevant data of enterprises and digital platforms. Then, descriptive statistical analysis is used to summarize the basic characteristics of the data, correlation analysis is used to test the relationship between variables, and hypothesis testing analysis is used to verify the research hypothesis. In addition, intermediary analysis and adjustment analysis are used to deeply understand the influence mechanism of digital platform on corporate environmental behavior and social responsibility. This paper fills the knowledge gap of the influence of digital platform on corporate environmental behavior and social responsibility, and provides practical and policy enlightenment. By deeply understanding the relationship between digital platform and sustainable development of enterprises, it can provide strong support for enterprises and governments to formulate more effective environmental protection policies and strategies.

There are three innovations in this paper. First, from the perspective of environmental science development, the influence mechanism of digital platform on corporate environmental behavior and social responsibility is deeply explored. The second is to put forward the application strategy of digital platform in corporate environmental behavior and social responsibility to provide guidance for corporate practice. Thirdly, by means of questionnaire survey, descriptive statistical analysis, correlation analysis and hypothesis test analysis, the influence mechanism of digital platform on corporate environmental behavior and social responsibility is comprehensively studied.

## 2 Literature review

Scholars have carried out extensive research in the field of corporate environmental behavior and CSR. They paid attention to the motivation, influencing factors and effects of corporate environmental protection behavior, and discussed the influence of CSR on corporate performance and sustainable development from different dimensions. Afsar and Umrani (11) investigated the influence of perceived CSR on employees’ environmental behavior. The results showed that perceived CSR had a significant and positive impact on environmental commitment. Raza et al. (12) investigated hotel employees’ views on CSR activities and their influence on employees’ voluntary environmental protection behavior based on the theory of social exchange and identity. The results showed that CSR had a direct impact on employees’ voluntary environmental protection behavior. Latif et al. (13) analyzed the relationship between CSR and employees’ environmental behavior from the perspective of sustainable development, and found that employees’ perceived CSR actively promoted employees’ environmental behavior. Deng et al. (14) studied the relationship between CSR initiatives in hospitals and employees’ environmental behavior, and found that CSR directly and indirectly affected employees’ environmental behavior through environment-specific transformational leadership. Guan et al. (15) proposed that CSR was mainly related to the environmental performance and economic performance of enterprises. Nowadays, people can improve the environmental performance and economic performance of enterprises by promoting employees’ environmental behavior and altruistic values, and realize CSR. Giacalone et al. (16) believed that CSR involved the aim of having a positive impact on the community operated by the analyzed company. International organizations and government agencies had also issued a series of environmental science guidelines to encourage enterprises to adopt sustainable development practices, reduce carbon emissions and protect ecosystems. The Global Environment Outlook report provided a comprehensive assessment of the global environmental situation, and called on governments, enterprises and all sectors of society to take actions to reduce carbon emissions, protect ecosystems and promote sustainable development. The report included detailed analysis and suggestions on many environmental problems such as climate change, biodiversity loss and land degradation, and encourages enterprises to take environmental protection measures to promote the realization of global sustainable development goals. It shows that the environmental protection behavior of enterprises has a far-reaching impact on their economic performance and social reputation. Environmental protection behavior not only helps to reduce the environmental footprint of enterprises, but also improves the trust of consumers and investors in enterprises. However, the environmental protection behavior of enterprises is influenced by many factors, including laws and regulations, market pressure and social expectations. Therefore, it has become an important topic to study how to promote enterprises to participate in environmental protection activities more actively.

The emergence of digital platform provides a new perspective for studying corporate environmental behavior and CSR. Among them, technologies and algorithms play a key role in the digital platform, which can be used for data analysis, user behavior prediction and information dissemination. The participation of artificial intelligence (AI) can effectively interact with experts and non-experts in different social places to promote the wise judgment of opaque artificial intelligence systems and realize their democratic governance (17). Li (18) believed that big data analysis played an important role in green governance and CSR. Kong and Liu (19) thought that digital transformation has greatly promoted CSR, and it was helpful to improve pollution control ability and internal control efficiency in enterprises with low financing constraints and high regulatory pressure, thus improving CSR performance. Li (20) evaluated the financial investment environment of enterprises based on blockchain and cloud computing, and found that cloud computing technology and blockchain technology expanded the construction performance of financial investment data from 5.98 to 9.27. The computing performance was improved by 3.29. Based on two-stage structural equation modeling-artificial neural network (ANN) method, Najmi et al. (21) discussed the role of consumers in the recycling plan of scrapped mobile phones. Yan et al. (22) used two-stage structural equation modeling and ANN to analyze the impact of the adoption of financial technology on the sustainable development performance of banking institutions. The research results showed that green finance and green innovation fully mediate the relationship between the application of financial technology and the sustainable development performance of banking institutions (22). Diaz and Nguyen (23) predicted the minimum prediction error of CSR index through gray correlation analysis and gray correlation analysis, and found that BPN model had the smallest prediction error, which was better than recurrent neural network (RNN) and radial basis function neural network model. Ezzi et al. (24) analyzed the important role of blockchain technology in explaining CSR performance, and the results showed that the implementation of blockchain technology had a significant and positive impact on CSR performance.

Wang et al. (25) constructed a recommendation and resource optimization model by using neural network algorithm from the perspective of cultural and creative industries to promote enterprise project decision-making and resource optimization. The research showed that the entrepreneurial project recommendation and resource optimization model can significantly improve the recognition accuracy, reduce the prediction error, and contribute to the sustainable development of social economy and the optimization of entrepreneurial resources. Combined with the research content of this paper, these research results can provide effective decision-making reference for enterprises and promote the realization of sustainable development goals. Wang et al. (26) used blockchain technology to build an intelligent contract, established a risk management system for online public opinion, and tracked public opinion through risk correlation tree technology, thus improving the accuracy of risk prediction and credibility detection. The research results showed that with the support of blockchain technology, the three experimental schemes designed can reasonably predict the risk and detect the credibility of NPO. This work was helpful to optimize the control measures of network environment and provide an important reference for improving the management level of network public opinion. Deng et al. (27) promoted the mechanism of public participation and enhanced the vitality of the economic market of resource-based cities by increasing policy intervention. This study had important reference value for promoting urban resource management and economic efficiency. Li et al. (28) paid attention to the influence of the pilot policy of low-carbon cities on urban entrepreneurial activities and its role in promoting green development. The results showed that the pilot policy of low-carbon cities generally inhibits entrepreneurial activities, but the level of green innovation can alleviate this inhibitory effect. In addition, the pilot policy of low-carbon cities inhibited the entrepreneurial activities of high-carbon industries, while encouraging the entrepreneurial activities of emerging industries, which led to the changes and upgrading of industrial structure. Li et al. (29) discussed the development path of clean energy and related issues of sustainable development of mining projects in the ecological environment driven by big data. Through this study, it was hoped to provide empirical support and decision-making reference for mining projects in the development of clean energy, promote the sustainable development of mining industry and realize a win-win situation of economic and ecological benefits. This was of great significance for protecting the ecological environment and realizing the sustainable utilization of resources. Li et al. (30) investigated the influence of regional digital finance development on corporate financing constraints. It was found that digital finance can significantly alleviate the financing constraints of enterprises, and the impact on small and medium-sized enterprises and private enterprises was more significant. Li et al. (31) discussed the impact of climate change on corporate environmental, social and governance performance. According to the empirical results, the environmental, social, and governance (ESG) performance of enterprises was significantly inhibited by climate change. It was also found that eliminating the mismatch between internal and external resources would help to alleviate the adverse impact of climate change on ESG performance.

The above literature review provides a comprehensive overview of the relevant research status and scholars’ views on corporate environmental behavior, CSR and digital platform. The research shows that scholars have carried out extensive research in the fields of corporate environmental behavior and CSR, and paid attention to different aspects of these fields, including environmental commitment, environmental behavior of employees, and sustainable development performance. Their research reveals the profound influence of environmental protection behavior of enterprises on their economic performance and social reputation, and the direct influence of CSR on employees’ voluntary environmental protection behavior. In addition, as a new technology and tool, digital platform has attracted the interest of research circles. Technologies and algorithms play a key role in the digital platform, which can be used for data analysis, user behavior prediction and information dissemination, thus affecting the environmental protection behavior and CSR of enterprises. Many studies have shown that AI, big data analysis, blockchain and other technologies have a positive impact on CSR performance and environmental protection behavior (32–35). However, these studies also have some limitations, such as differences in research methods, limitations in sample selection and heterogeneity between different fields. Therefore, this paper aims to explore the influence mechanism of digital platform on corporate environmental behavior and social responsibility, adopt various research methods, and pay attention to the differences between different industries and geographical regions. This will help to fill the knowledge gap in existing research and provide more specific guidance for enterprises and policy makers to promote the realization of sustainable development goals.

The design of this paper focuses on the interaction between digital platform and corporate social responsibility and its influence on environmental protection behavior, which reflects the complexity and scientific value of the study. Based on the theoretical framework and previous empirical research, this paper investigates how the digital platform affects the environmental protection behavior by promoting the practice of corporate social responsibility. This not only deepens the understanding of the role of digital platform in the field of corporate social responsibility, but also provides a new perspective on how to use digital technology to promote environmentally friendly behavior of enterprises, thus filling the gaps in the existing literature.

## 3 Research methodology

### 3.1 Cross-influence of CSR and development of environmental science

CSR and environmental science development are two interrelated fields, and their cross-influence is very important for understanding the mechanism behind corporate environmental protection behavior. This section deeply discusses the relationship between CSR and the development of environmental science, and establish the theoretical basis of the research. In this section, the guiding principles of environmental science development, including environmental protection and sustainable development policy documents issued by international organizations such as the United Nations Environment Programme and government agencies, are shown in Table 1.

**Table 1:** Guidance document for the development of environmental science.

Document name	Document content	Document publishing organization
2030 Agenda for Sustainable Development	Sustainable Development Goals (SDGs) in 17 aspects including economy, society and environment. It aims to solve global challenges, including poverty, climate change and ecosystem deterioration	United Nations sustainable development agenda
The Global Environment Outlook Attached with Global Environment Outlook Policy Options	Analysis of environmental problems and policy suggestions	United Nations Environment Programme (UNEP)
World Energy Outlook Renewable Energy Outlook	Sustainable energy development and carbon emission reduction	International Energy Agency (IEA)
Clean Energy Plan Water Resource Protection Plan	Protect the environment and promote sustainable development	United States Environmental Protection Agency

In Table 1, the common goal of core policies and plans is to encourage enterprises to adopt sustainable development practices, reduce carbon emissions and protect ecosystems, thus promoting global sustainable development. Enterprises can fulfill their social and environmental responsibilities by actively participating in these initiatives and complying with relevant policies. Meanwhile, they can gain economic and reputation benefits in terms of sustainability. These policies and plans provide a framework and guidance for enterprises to play an active role in environmental protection behavior (36, 37).

**Figure 1:**
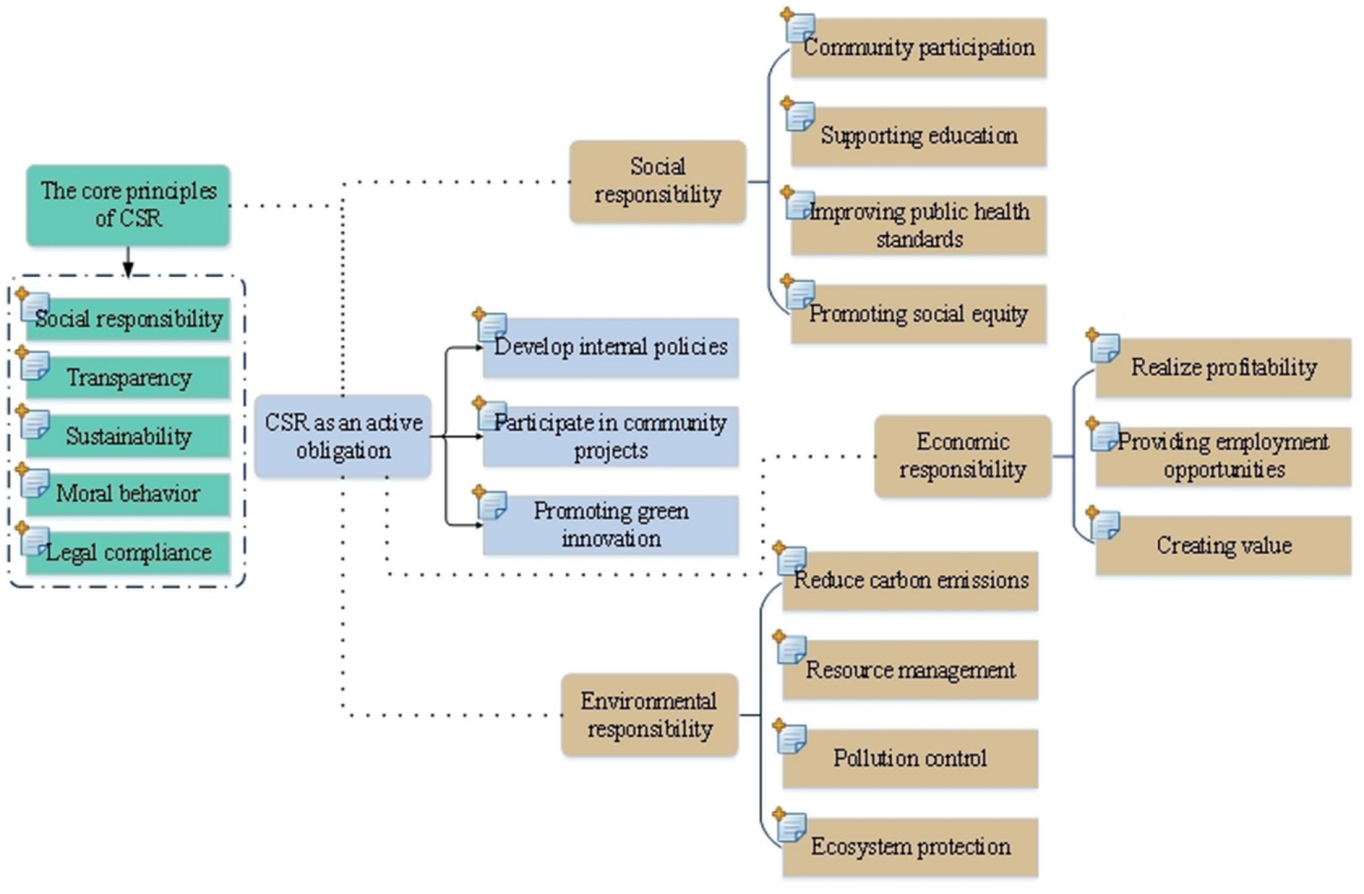
Cross-influence of CSR and the development of environmental science.

CSR covers the social and environmental impacts of enterprises in their business activities, and emphasizes the active obligations of enterprises in fulfilling their social responsibilities (38). Figure 1 shows the cross influence of CSR and the development of environmental science.

In Figure 1, in the cross-influence between CSR and environmental science, the core principles and active obligations of CSR play a key role. The core principles of CSR, such as social responsibility, transparency and sustainability, guide enterprises to actively consider social and environmental factors in their business activities. Meanwhile, CSR, as an active obligation, requires enterprises not only to fulfill their legal obligations, but also to actively participate in solving social and environmental problems. These behaviors are the concrete application of CSR in the environmental field, which shows how enterprises actively fulfill their social and environmental responsibilities and promote the practice of sustainable development.

### 3.2 Potential mechanism of digital platform in enterprise environmental protection behavior

**Figure 2:**
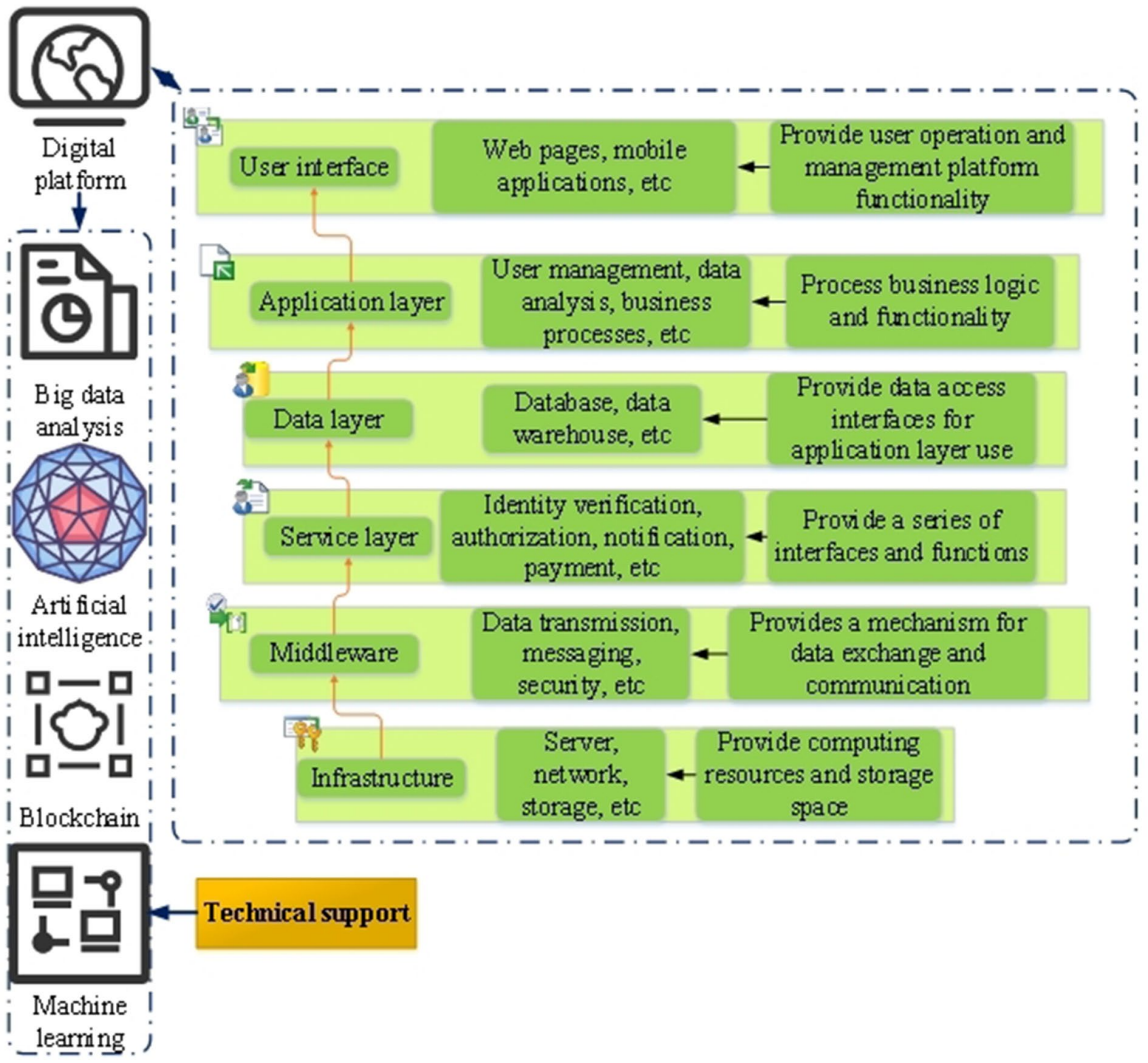
Digital platform technology architecture.

Digital platform refers to a platform based on digital technology and Internet, which connects different participants and provides various services and solutions through online interaction and data sharing (39). Figure 2 shows the technical architecture of digital platform.

**Figure 3:**
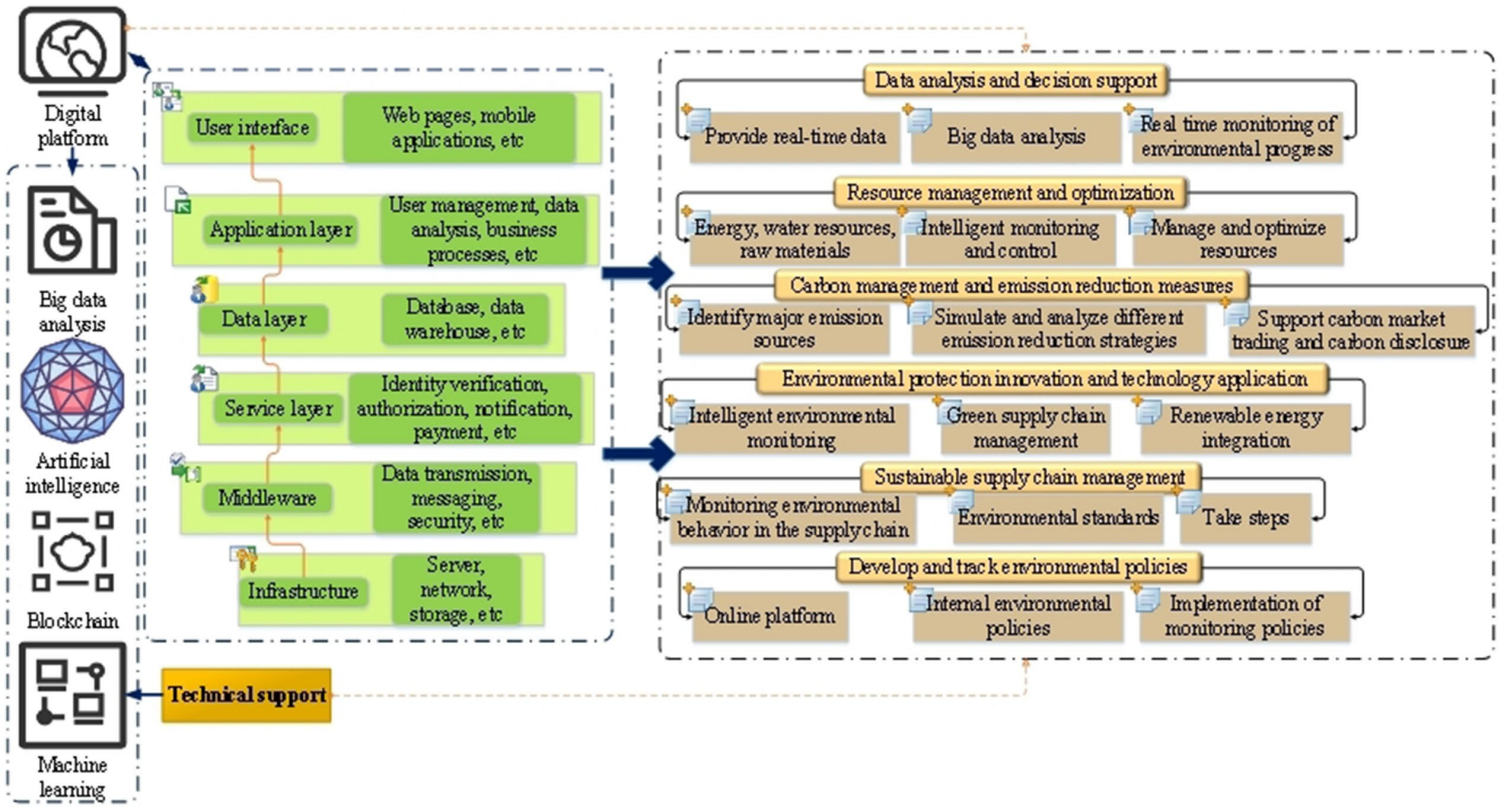
Potential mechanism of digital platform in enterprise environmental protection behavior.

The technical architecture of Figure 2 digital platform includes infrastructure, middleware, service layer, data layer, application layer and user interface. The user interface is the part where users interact with the digital platform, which provides the functions of users to operate and manage the platform. The application layer is responsible for handling business logic and functions. The data layer is responsible for data storage, reading, updating and deleting, and provides data access interfaces for the application layer. The service layer is a part that provides various services, and provides a series of interfaces and functions for the application layer to call and use. Middleware is a part that connects various components and levels, provides a mechanism for data exchange and communication, and ensures the coordination and interaction between various parts. Infrastructure provides computing resources and storage space to ensure the stability and reliability of the digital platform (40, 41). Figure 3 shows the potential mechanism of digital platform in enterprise environmental protection behavior.

In Figure 3, digital platform plays an important role in corporate environmental behavior. Through data analysis, resource management, carbon management, environmental protection innovation and other mechanisms, the digital platform helps enterprises to better fulfill their social and environmental responsibilities, promote the practice of sustainable development, improve the environmental performance of enterprises, create economic value for enterprises, and promote the positive relationship between environmental protection and sustainability (42).

### 3.3 Research hypothesis

The research hypothesis is a speculative statement about the relationship among different variables. The research hypothesis of this paper focus on the influence of digital platform on corporate environmental behavior and social responsibility.

*Hypothesis 1*: There is a positive correlation between the widespread application of digital platforms and corporate environmental protection behavior.

*Hypothesis 2*: There is a positive correlation between the environmental protection innovation technology of digital platform and the implementation of environmental protection policies of enterprises.

*Hypothesis 3*: There is a positive correlation between social responsibility tools of digital platform and CSR activities.

*Hypothesis 4*: There is a positive correlation between enterprise scale and industry type on digital platform and enterprise environmental behavior.

### 3.4 Method of data capture

In this study, the questionnaire design is to explore the influence of digital platform on corporate social responsibility practice by investigating employees in private enterprises. In order to ensure that the questionnaire can accurately reflect the actual digital actions and CSR activities of enterprises, a series of measures have been taken to enhance the reliability and validity of the questionnaire. Firstly, before designing the problem, the relationship between CSR and the development of environmental science is deeply studied, and the cross influence of CSR and environmental science is clarified. With reference to the policy documents on environmental protection and sustainable development issued by international organizations such as the United Nations Environment Programme, the theoretical basis of the research is constructed. This helps to ensure that the questionnaire design is closely related to the research objectives. Secondly, in the process of questionnaire design, 20 professionals with relevant backgrounds are invited to fill in the first edition of the questionnaire, and the expression and order of questions are adjusted according to their feedback to improve the clarity and logic of the questionnaire. This step is helpful to optimize the questionnaire design, ensure that the questions are accurate and clear, and capture the required information effectively. In addition, referring to the published related research, a measurement tool is constructed based on the indicators used in these studies to ensure the relevance and effectiveness of the questionnaire. In order to further improve the reliability and representativeness of the questionnaire, the online survey platform is used to distribute the questionnaire, and a reminder mechanism is set up to increase the response rate. Meanwhile, small rewards are provided for participants who completed the questionnaire to ensure the data quality. Cronbach’s α coefficient and exploratory factor analysis are used to verify the internal consistency test of sample data to evaluate the consistency and reliability of the questionnaire results. In addition, Pearson correlation coefficient is used to evaluate the correlation among different variables to ensure the accuracy and reliability of data analysis. In the questionnaire design, the respondents of private enterprises are divided into three categories: managers, team members and ordinary employees to ensure that employees with different positions and responsibilities are covered to fully understand the digital actions and CSR activities of enterprises. Through the questionnaire collection and analysis of employees in different positions, people can better understand the views and practices of digital platforms and environmental protection behaviors at all levels within the enterprise, and thus draw more objective research conclusions. The comprehensive application of the above measures makes it possible to explore the influence of digital platform on corporate social responsibility practice more comprehensively and accurately, and ensure that the obtained data has high credibility and representativeness, thus providing a solid foundation for subsequent analysis and conclusions. The specific questionnaire design and collection contents are as follows:

The choice of questionnaire survey in this paper is mainly based on its ability to effectively collect a wide range of data, while ensuring anonymity and authenticity. Compared with other data collection methods, questionnaire survey can cover a wider audience and get direct feedback on their opinions and behaviors, which is very important for exploring the role of digital platform in corporate environmental protection behavior.

In this paper, the data of environmental behavior and environmental science development released by the United Nations Environment Programme are used as the control data set of questionnaire survey results. Questionnaire survey is the main means to obtain information about environmental behavior and social responsibility of participating enterprises. Siyal et al. (43) used questionnaires to analyze how inclusive leaders cultivate employees’ innovative work behavior and creativity, and the results showed that inclusive leadership had a positive impact on innovative work behavior and creativity. In this paper, the respondents of private enterprises are divided into three categories: managers (M) who are related to environmental protection behavior and social responsibility activities of enterprises, team members (T) who are responsible for social responsibility, and ordinary employees (N). The sample size is determined based on Cochran formula. Considering the expected effect, α level and statistical power, it is estimated that at least 250 questionnaires are needed to ensure the reliability and representativeness of the research results. Finally, 256 valid questionnaires are collected, which meets the demand of sample size. After the preliminary design of the questionnaire, 20 professionals with relevant backgrounds are invited to fill it out, and the expression and order of the questions are adjusted based on their feedback to improve the clarity and logic of the questionnaire.

In order to ensure the validity and reliability of the questionnaire, this paper refers to the published related research and builds a measurement tool based on the indicators used in these studies. By using the online survey platform to distribute questionnaires and setting up a reminder mechanism, the response rate is improved, and small rewards are provided to participants who complete the questionnaires to ensure the data quality. In order to verify the consistency and reliability of data, Cronbach’s α coefficient and exploratory factor analysis are used for internal consistency test, and Pearson correlation coefficient is also used to evaluate the correlation among variables. The questionnaire is distributed to 297 respondents by e-mail or online survey platform. Two hundred and fifty six valid questionnaires are collected.

The questionnaire is divided into six sections. The first section is basic information statistics, including gender, working years, education level and occupation. The second section is the development level of environmental science, which mainly focuses on the degree of attention paid by enterprises to the development of environmental science and whether enterprises are developing or applying related technologies of environmental science. The third section is the application level of digital platform, knowing the application of digital platform in the enterprise where the interviewee works, including: the experience of using digital platform, whether the enterprise widely uses digital platform to support business operations, and whether the enterprise uses digital platform to monitor and manage data related to environmental protection and social responsibility. The fourth section is the environmental behavior of enterprises, mainly including whether enterprises have taken measures to reduce carbon emissions and whether enterprises actively participate in resource management and sustainable practice. The fifth section investigates the respondents’ questions about CSR activities, and whether they hold positions related to environmental protection or social responsibility, including: whether enterprises actively participate in social responsibility activities, such as charitable donations and community support. Whether the enterprise has social responsibility report or traceable social responsibility record. The sixth section is the intermediary role of digital platform in environmental behavior and social responsibility, mainly including whether enterprises use digital platform to monitor and report environmental behavior and social responsibility activities. In the definition of variables and the construction of measurement scale, this paper defines “corporate social responsibility” as that enterprises voluntarily assume social and environmental responsibilities while pursuing economic benefits. “Digital platform usage” refers to the degree to which enterprises integrate and use digital technology platforms in their operations and management. “Environmental protection behavior” covers all practical actions taken by enterprises to reduce environmental impact and promote sustainable development. The measurement of these variables is based on the previous literature review, combined with expert opinions and pretest results, forming a set of scales containing multiple items, aiming at comprehensively and accurately capturing the core content of each variable. Table 2 shows the definition and selection basis of research variables:

**Table 2:** Study the definition and selection basis of variables.

Variable name	Definition	Selection basis
Digital platform usage	The frequency and depth of enterprises’ application of digital platforms in their daily operations	According to the existing literature, the wide application of digital platform is considered to be an important factor affecting the environmental behavior of enterprises
Corporate environmental protection behavior	Include measures to reduce carbon emissions, manage resources and adopt sustainable practices	Environmental protection behavior is a key aspect to measure the practice of corporate social responsibility, and it also directly reflects the response of enterprises to the development guide of environmental science
Application of social responsibility tools	CSR activities implemented by enterprises through digital platforms, such as charitable donations and community support	Social responsibility tool is a means for enterprises to fulfill their social responsibilities, which is helpful to promote the positive role of enterprises in the field of public health
Environmental innovation technology	New technologies applied by enterprises to improve the efficiency and effect of environmental protection	Environmental innovation technology is regarded as the key driving force to promote environmental protection behavior and sustainable development of enterprises

According to the intermediary effect analysis method mentioned by Alfons et al. (44), Pearson correlation coefficient and Bootstrap method are used in this paper to evaluate the relationship among digital platform usage, CSR policy implementation and corporate environmental behavior. This method is widely recognized and used in social science research, and has been recognized by academic circles for its robustness and applicability. Pearson correlation coefficient is used to analyze the correlation among different variables, and the calculation is shown in Equation (1):


(1) $$r=\frac{n\left(\sum xy\right)-\left(\sum x\right)\left(\sum y\right)}{\sqrt{\left[n\sum x^{2}-{\left(\sum x\right)}^{2}\right]\;\left[n\sum y^{2}-{\left(\sum y\right)}^{2}\right]}}$$

In Equation (1), r represents the correlation coefficient. x and y represent two variables respectively, and n represents the sample size. Using Baron and Kenny’s mediation effect analysis method, Equations (2–4) shows the calculation of intermediary effect:


(2) $$a=\frac{X-M}{X}$$

(3) $$b=\frac{Y-X}{Y-M}$$

(4) $$c^{\prime }=\frac{Y-M}{X}$$

In the above equations, a stands for total effect, b stands for direct effect, $c^{\prime }$ stands for indirect effect, X stands for intermediary variable (application level of digital platform), M stands for the influence of intermediary variable on dependent variable, and Y stands for dependent variable (environmental protection behavior or social responsibility activities of enterprises).

## 4 Results and discussion

### 4.1 The results of reliability and validity test and descriptive statistical analysis of the questionnaire

**Figure 4:**
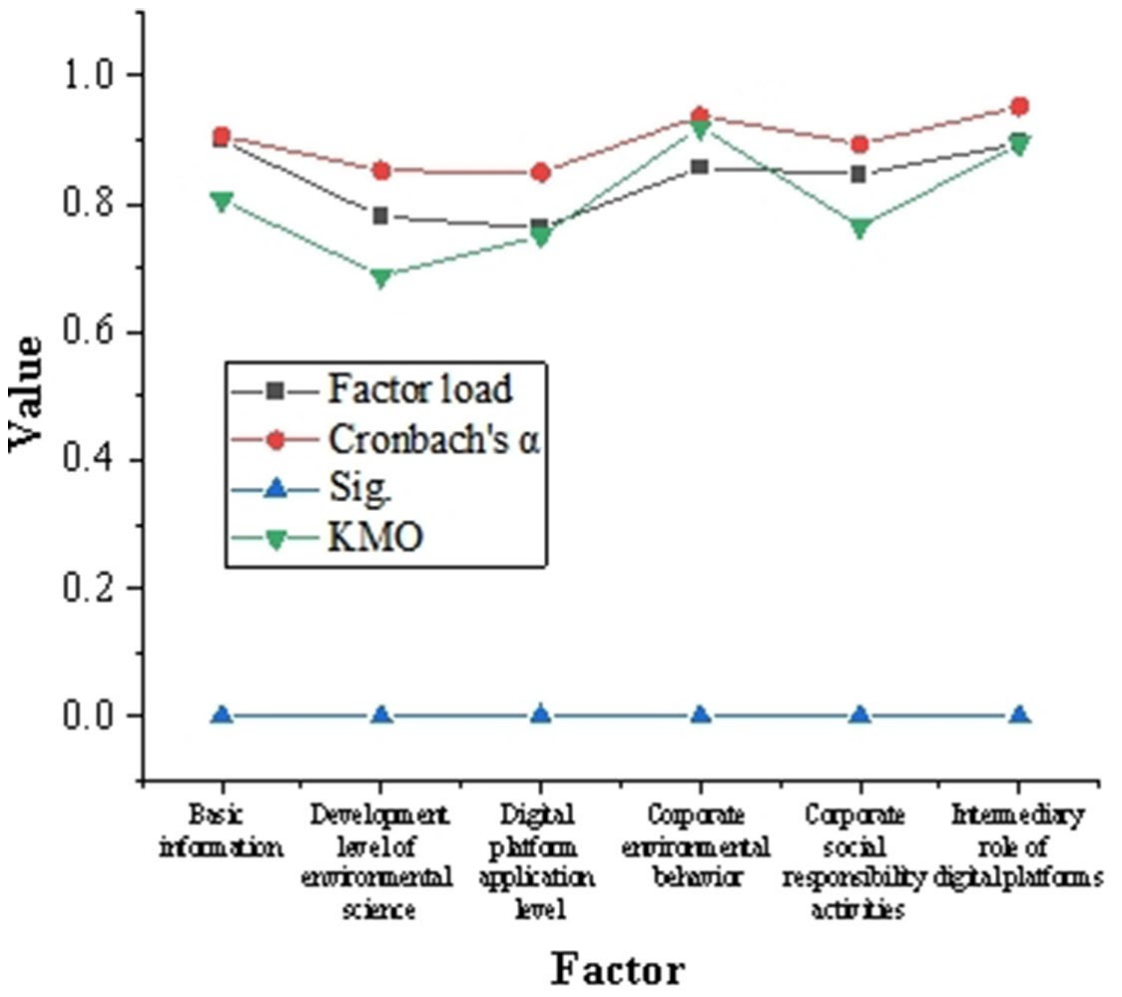
Results of reliability and validity test of questionnaire.

The reliability and validity of the questionnaire are shown in Figure 4. It shows that each factor has a high reliability coefficient (greater than 0.84), the factor load (greater than 0.75) indicates that there is a correlation between the problem and each factor, and the KMO value shows that the data is applicable in factor analysis.

**Figure 5:**
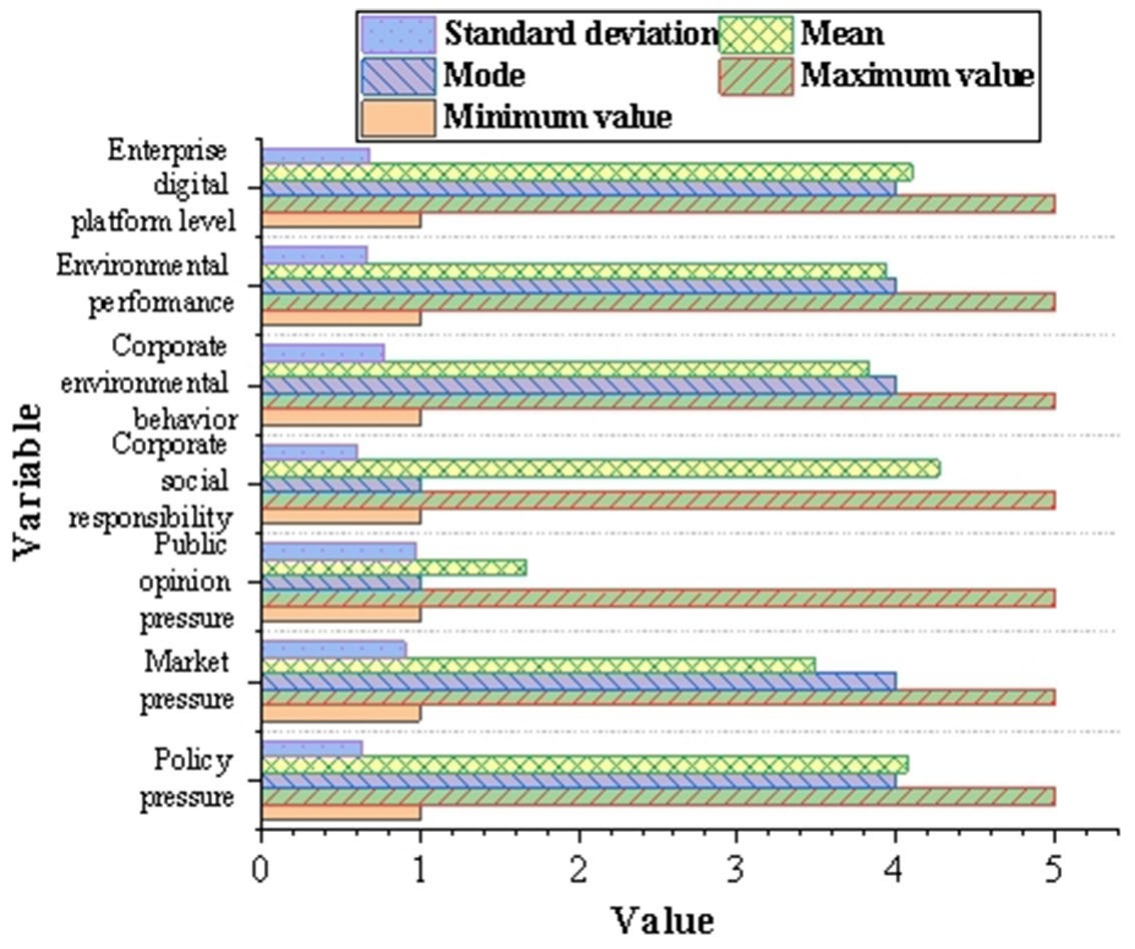
Descriptive statistical analysis results of the questionnaire.

Figure 5 shows the descriptive statistical analysis results of the questionnaire. According to the descriptive statistical results, the respondents’ average scores on policy pressure, market pressure, CSR, environmental performance, and enterprise digital platform level are 4.07, 3.49, 4.27, 3.93, and 4.1, respectively. The evaluation results are relatively consistent. However, there are great differences in the evaluation of public opinion pressure and corporate environmental protection behavior.

### 4.2 The correlation between the usage of digital platform and the environmental protection behavior of enterprises

**Figure 6:**
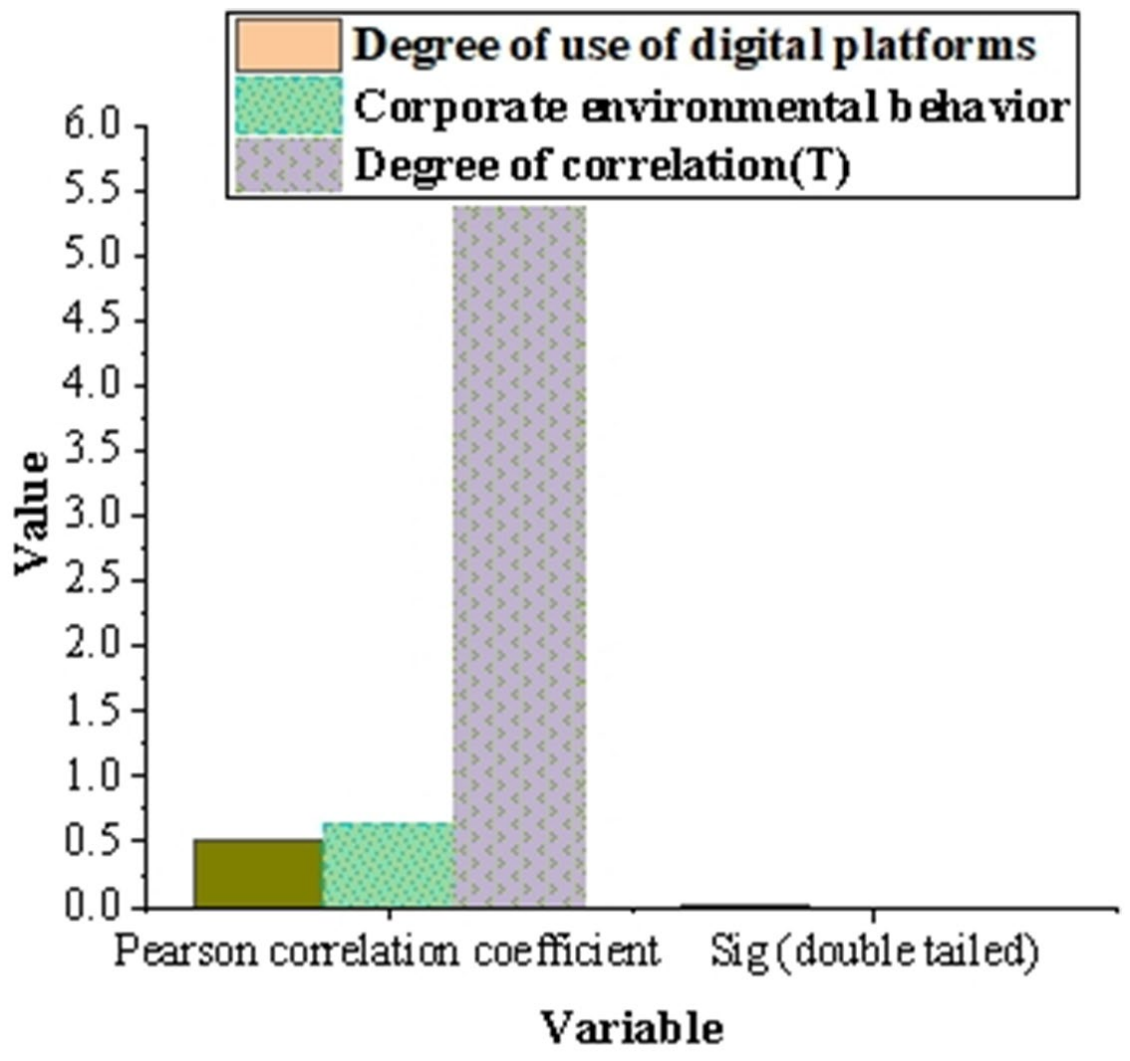
The results of correlation analysis between the use of digital platform and the environmental protection behavior of enterprises.

Figure 6 shows the results of correlation analysis between the usage of digital platform and the environmental protection behavior of enterprises. Pearson correlation coefficient shows that there is a moderate positive correlation between the use of digital platforms and corporate environmental behavior (correlation coefficient is 0.523). The Sig. value of correlation analysis is 0.001 (<0.05), which indicates that this correlation is significant. The correlation between the usage of digital platform and enterprise’s environmental behavior is 5.367, Sig. = 0.000 (*p* < 0.05), which verifies hypothesis 1.

**Figure 7:**
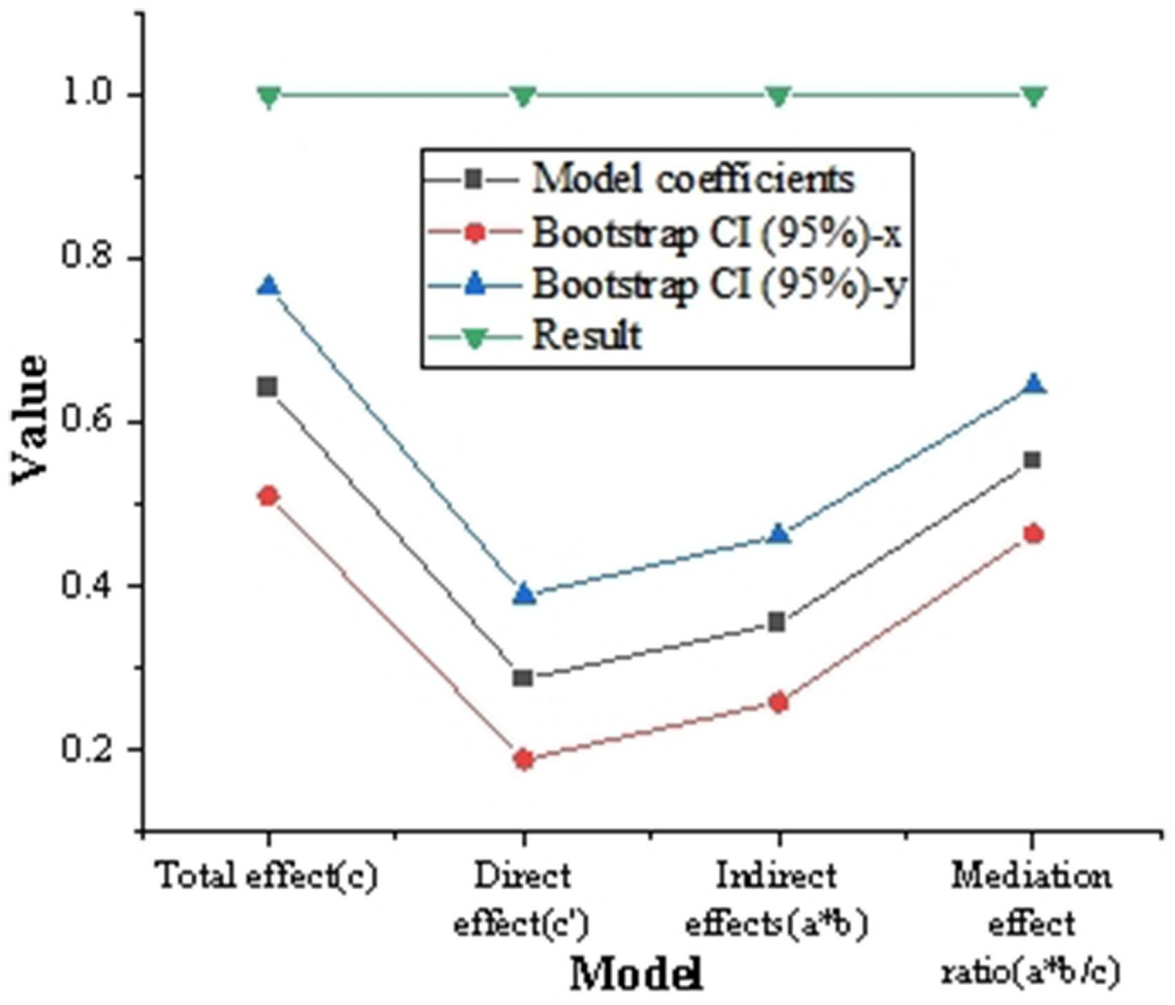
Intermediary analysis of the usage degree of digital platform.

Figure 7 shows the intermediary analysis of the usage of digital platform. The intermediary analysis shows that the intermediary effect ratio (a * b/c) is 55.31%, and the 95% Bootstrap CI range does not include 0, which indicates that the usage of digital platform plays a significant intermediary role between digital platform and corporate environmental protection behavior.

### 4.3 The influence of digital platform on CSR policy and practice

**Figure 8:**
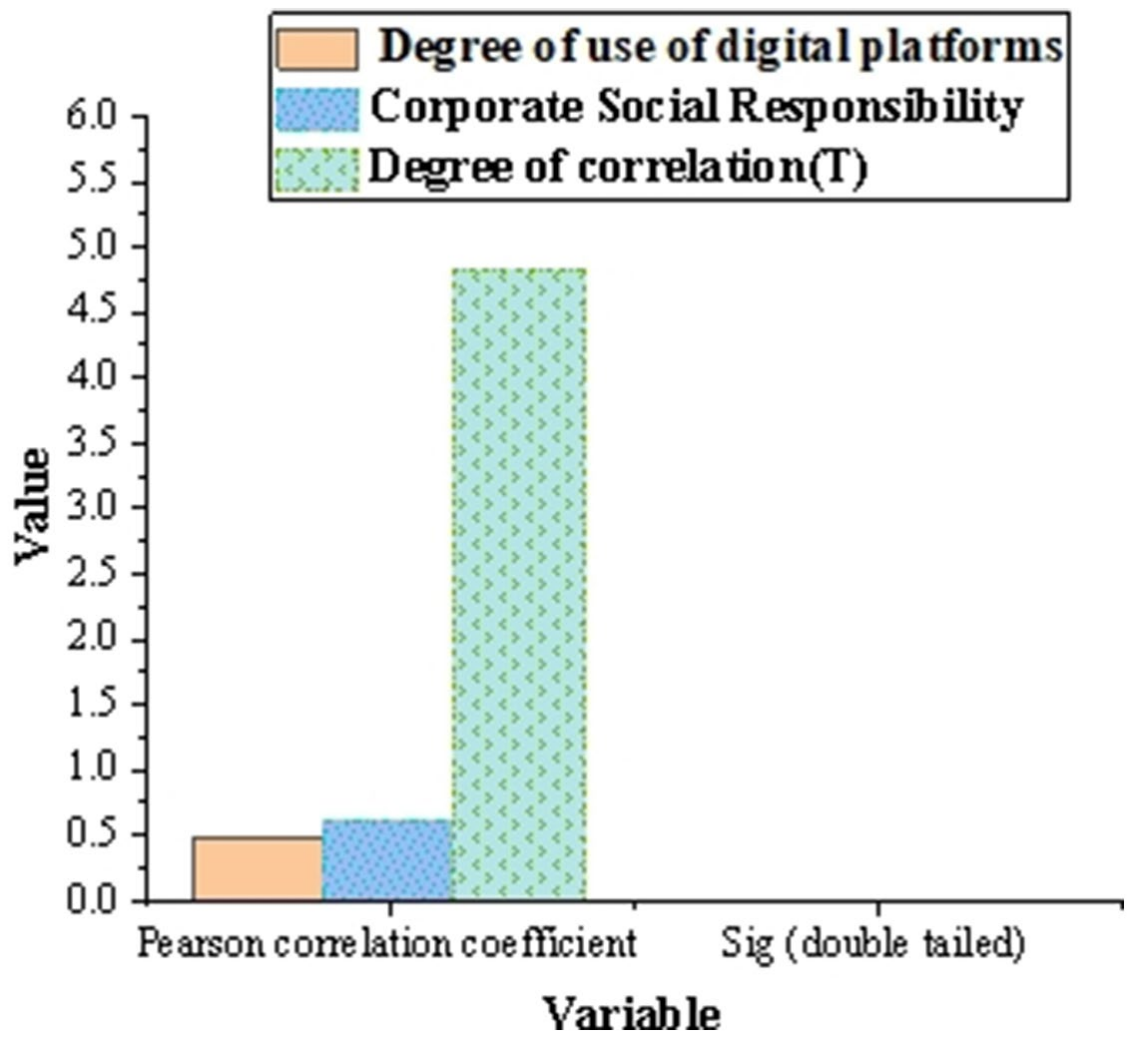
Correlation analysis results between digital platform and CSR.

Figure 8 shows the results of correlation analysis between digital platform and CSR. Pearson correlation coefficient shows that there is a moderate positive correlation between the use of digital platforms and CSR policies and practices (correlation coefficient is 0.481). The Sig. value of correlation analysis is 0.003, which is less than the significance level of 0.05, indicating that this correlation is significant. The correlation T between digital platform and CSR is 4.825, Sig. = 0.000 (*p* < 0.05), which shows that there is a positive correlation between digital platform’s social responsibility tools and CSR activities, and supports hypothesis 3.

**Figure 9:**
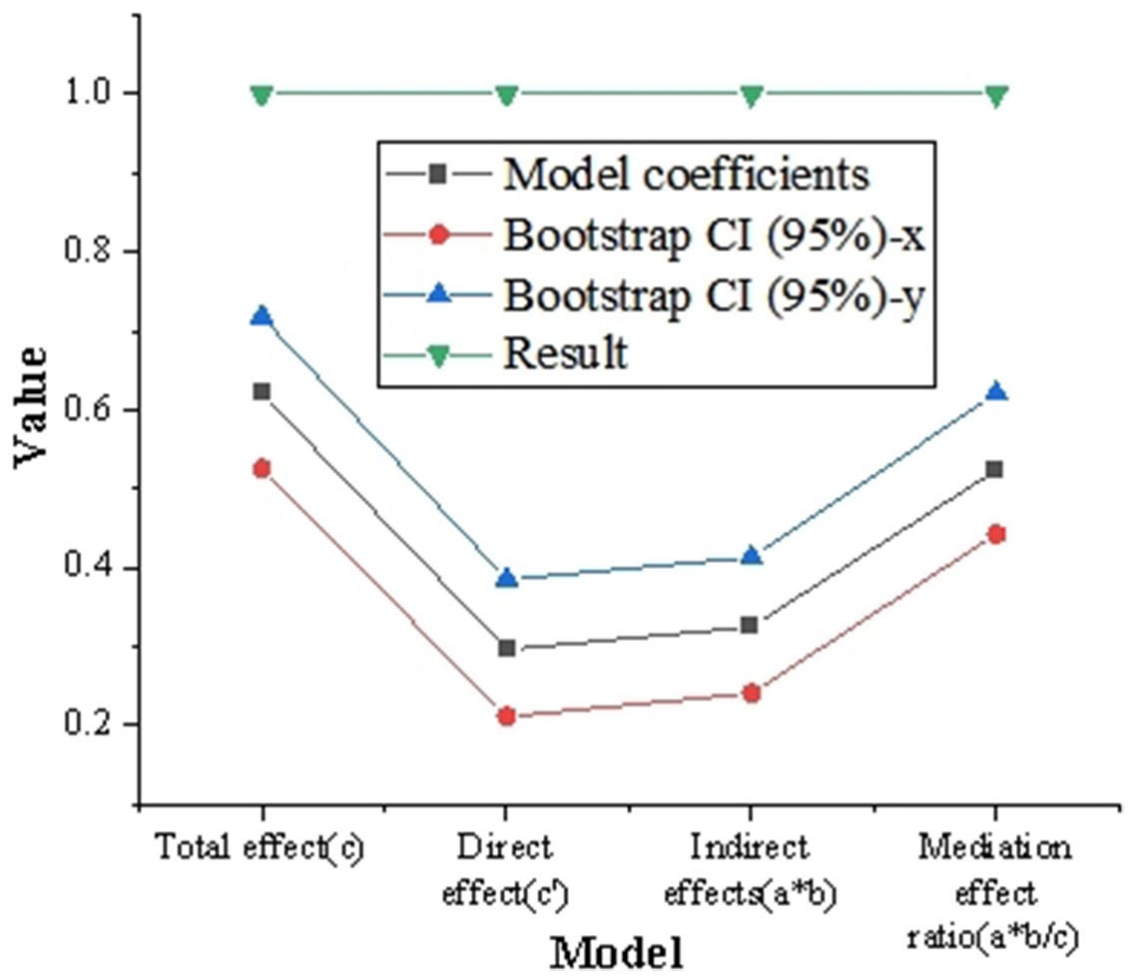
Intermediary analysis of digital platform on CSR policy and practice.

Mediating analysis shows that the mediating effect ratio (a * b/c) is 52.40%, and the 95% Bootstrap CI range does not include 0, indicating that the usage of digital platforms plays a significant mediating role between digital platforms and CSR policies and practices. Figure 9 shows the intermediary analysis of digital platform on CSR policy and practice.

### 4.4 Mediating and regulating functions of digital platform and enterprise’s environmental protection behavior

**Figure 10:**
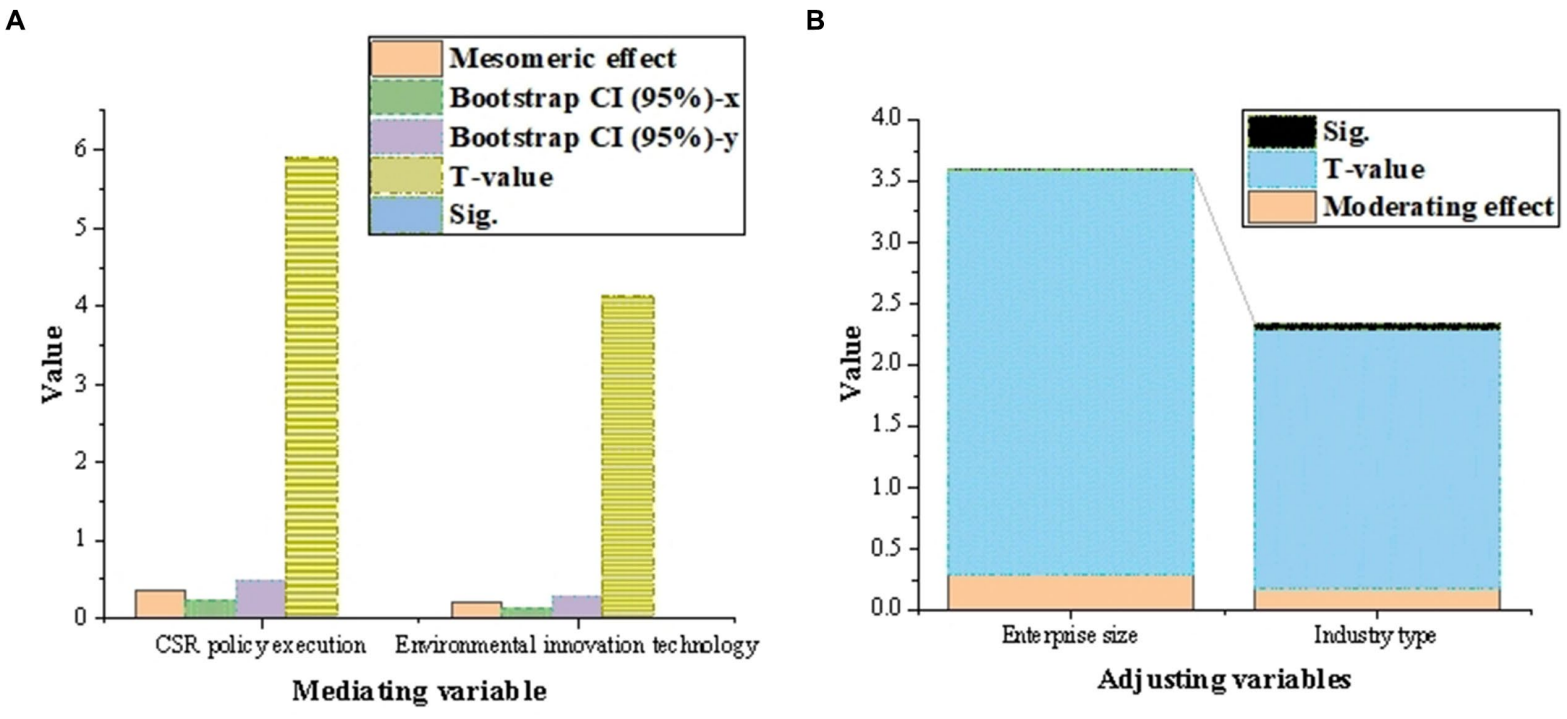
The analysis results of the mediating and regulating effects of digital platform on enterprise’s environmental protection behavior [**(A)** the mediating effect; **(B)** for regulatory purposes].

Figure 10 shows the analysis results of the intermediary role and regulatory role of digital platform on enterprise environmental protection behavior. The total effect (a) of digital platform on corporate environmental behavior is 0.627, the total effect (b) of intermediary variable CSR policy implementation is 0.452, and the total effect (b) of intermediary variable environmental innovation technology is 0.313. The mediating effect and 95% confidence interval calculated by Bootstrap method show that the mediating variable CSR policy implementation and environmental protection innovation technology significantly mediate the influence of digital platform on corporate environmental protection behavior, because their confidence intervals do not include 0. *T*-value and Sig. value also support the significance of these mediating effects. The moderating effect of moderating variable enterprise scale is 0.284, and that of moderating variable industry type is 0.179. The *t*-value and Sig. value of the regulatory effect show that both the scale of enterprises and the types of industries have a significant regulatory effect on the impact of digital platforms on corporate environmental behavior.

In order to further explore the potential causal relationship between the use of digital platforms and the environmental behavior of enterprises, Structural Equation Modeling (SEM) is introduced for analysis. In addition, through the analysis of mediating and moderating effects, it further analyzes how the digital platform affects the CSR practice and environmental behavior of enterprises through different mediating variables (environmental innovation technology) and moderating variables (enterprise scale and industry type). Firstly, a structural equation model is established to evaluate the direct and indirect relationship between digital platform use (independent variable) and enterprise environmental behavior (dependent variable). As a part of indirect relationship, two intermediary variables are considered: CSR policy implementation and environmental innovation technology. Meanwhile, enterprise scale and industry type are regarded as moderating variables to test whether they will change the correlation between the main variables. The hypothesis is tested by multiple regression analysis. This analysis helps to verify the correlation between the use of digital platform, the implementation of CSR policy, environmental innovation technology and corporate environmental behavior, and also examines the regulatory role of enterprise scale and industry type. Table 3 shows the results of multiple regression analysis, which is used to test the direct impact of the use of digital platforms on corporate environmental behavior and its indirect impact through intermediary variables.

**Table 3:** Results of SEM and multiple regression analysis.

Variable	Coefficient ( β )	Standard error	*T* value	*p*-value
Use of digital platform	0.623	0.041	15.21	<0.001
Implementation of CSR policy	0.457	0.038	12.03	<0.001
Environmental innovation technology	0.342	0.045	7.60	<0.001
Enterprise scale (adjusting variable)	0.198	0.052	3.81	<0.001
Industry type (adjustment variable)	0.162	0.055	2.95	0.003

*p* < 0.05 indicates that the result is statistically significant.

In Table 3, the use of digital platform has a significant positive impact on corporate environmental behavior (β = 0.623, *p* < 0.001), and CSR policy implementation and environmental innovation technology both show significant positive effects as intermediary variables. In addition, as moderating variables, enterprise scale and industry type have a significant moderating effect on the relationship between the main variables. Through the structural equation model and the results of multiple regression analysis, it is confirmed that there is a significant positive relationship between the use of digital platforms and corporate environmental behavior. Environmental innovation technology and the implementation of CSR policy have played an important intermediary role in this relationship. In addition, the analysis also reveals the moderating role of enterprise scale and industry type in the relationship between digital platform use and enterprise environmental behavior. This emphasizes the need to consider the specific background and characteristics of enterprises when encouraging enterprises to take digital measures to improve their environmental performance. The above findings have important implications for decision makers and policy makers. They emphasize the necessity of supporting enterprises to adopt digital technology to improve environmental protection behavior and CSR practice, and suggest the importance of considering enterprise scale and industry characteristics when designing relevant policies and interventions.

The findings of this paper provide valuable insights for decision makers and policy makers. Firstly, the paper emphasizes the core role of digital platform in promoting corporate environmental behavior and social responsibility practice. The application of digital technology can help enterprises to manage resources more efficiently and formulate environmental protection strategies, thus promoting sustainable development. It is suggested that policy makers should support and encourage enterprises to adopt digital technology to improve their environmental friendliness and social responsibility practice. Secondly, future policy planning needs to take into account the differences in the influence of enterprise scale and industry type on digital platforms. Enterprises of different scales and industries may face different challenges and opportunities in digital transformation, so customized guidelines are needed to guide them to make rational use of digital platforms. Policymakers can formulate targeted policies and measures according to the characteristics of different enterprises to promote the combination of digitalization and sustainable development. Finally, it is suggested that further research should pay attention to the differences in the impact of digital platforms on corporate social responsibility and public health in different regions and cultural backgrounds. Different regions and cultures may have different degrees of acceptance and practice of digitalization, which will have different degrees of impact on corporate social responsibility and public health. In-depth study of the mechanism of digital platforms in different contexts will help to better guide enterprises and policy makers in their decision-making and practice in different environments. Through these suggestions and research directions, people can better promote the goals of corporate social responsibility and sustainable development with the help of digital platforms.

## 5 Conclusion

The purpose of this paper is to explore the influence of digital platform on corporate environmental behavior and social responsibility, and to deeply understand how digital platform shapes the sustainable development practice of enterprises. Through comprehensive analysis of questionnaire survey data and various research methods, it is found that digital platform plays an active role in the sustainable development of enterprises. There is a positive correlation between the wide application of digital platform and corporate environmental behavior and social responsibility, which shows that digital platform helps enterprises to participate in environmental protection and social responsibility activities more actively and promote sustainable development. Secondly, the environmental protection innovation technology of digital platform has a positive impact on the implementation of environmental protection policies of enterprises. Environmental protection innovation technology plays an intermediary role between digital platform and enterprise environmental protection behavior, which strengthens the influence of digital platform on enterprise environmental protection behavior. In addition, the scale of enterprises and the types of industries plays a regulatory role in the influence mechanism of digital platforms. Enterprises of different scales and industries have different responses to digital platforms, which requires individualized consideration when formulating environmental protection policies and strategies. However, there are some shortcomings in this paper. The research sample has limitations and may not fully represent enterprises of other industries and scales. Future research can expand the sample range, deeply analyze the relationship between digital platform and sustainable development of enterprises, and consider more regulatory factors.

## Data availability statement

The original contributions presented in the study are included in the article/supplementary material, further inquiries can be directed to the corresponding author.

## Author contributions

MW: Conceptualization, Data curation, Validation, Writing – review & editing. RY: Conceptualization, Formal analysis, Writing – original draft. XG: Investigation, Methodology, Writing – original draft. ZW: Formal analysis, Methodology, Visualization, Writing – review & editing. YZ: Investigation, Software, Writing – review & editing. TL: Funding acquisition, Project administration, Resources, Software, Supervision, Writing – original draft.
